# MiRNA-149 modulates chemosensitivity of ovarian cancer A2780 cells to paclitaxel by targeting MyD88

**DOI:** 10.1186/s13048-015-0178-7

**Published:** 2015-07-30

**Authors:** Yueping Zhan, Fenfen Xiang, Rong Wu, Jian Xu, Zhenhua Ni, Jiemin Jiang, Xiangdong Kang

**Affiliations:** Department of Central Lab, Putuo Hospital, Shanghai University of Traditional Chinese Medicine, Shanghai, 200062 P.R. China; Department of Laboratory Medicine, Putuo Hospital, Shanghai University of Traditional Chinese Medicine, Shanghai, 200062 P.R. China

**Keywords:** miRNA-149, MyD88, Paclitaxel, Chemosensitivity, Ovarian cancer

## Abstract

**Background:**

The low effectiveness of anticancer drugs remains a major unresolved obstacle to successful chemotherapy. Recently, much evidence on the roles of miRNAs in determining drug-sensitivity/resistance has been emerging. The relationship between miRNA-149 expression and paclitaxel chemoresistance in human ovarian cancer cells remains largely unknown.

**Methods:**

This study investigated the relationship between miRNA-149 expression and the sensitivity of ovarian cancer A2780 cells to paclitaxel treatment. To achieve the down-regulation of miRNA-149 gene expression in A2780 cell line, the cells were infected with lentivirus carrying inhibitor of miRNA-149. Western blot and qRT-PCR were used to detect relevant protein levels and the expressions of mRNAs of interest. Cell proliferation was measured by CCK-8 assay. Flow cytometry was used to measure cell cycle and apoptosis. Transwell migration assay was used to observe the change of migration of transfected cells.

**Results:**

Down-regulation of miRNA-149 decreased the sensitivity of ovarian cancer A2780 cells to paclitaxel. After paclitaxel treatment, decreased apoptosis and G2 phase ratio, increased cell migration, increased level of Bcl-2, and decreased level of Bax were found in miRNA-149-down-regulated A2780 cells. MiRNA-149 down-regulation resulted in increased expression of MyD88 in A2780 cells. Down-regulation of miRNA-149 in A2780 cells increased MyD88 expression and decreased their sensitivity to paclitaxel treatment.

**Conclusion:**

Our findings suggest that miRNA-149 mediates the susceptibility of paclitaxel by regulating MyD88 expression in ovarian cancer cells.

## Introduction

Ovarian cancer is a common tumor and the most lethal malignancy of the female reproductive organs. A combination of carboplatin and paclitaxel has been widely used as chemotherapy for ovarian cancer patients. Although the patients initially respond successfully to paclitaxel-based chemotherapy, in most cases, they eventually become insensitive to the chemotherapy [1]. Several mechanisms have been demonstrated regarding chemoresistance to paclitaxel, such as over-expression of the multidrug transporter P-glycoprotein [2], selective expression of beta-tubulin isotypes [3], down-regulation of bcl-2 [4], or aberrant cell signaling [5]. Nevertheless, the overall molecular mechanisms of paclitaxel resistance have yet to be clarified.

MicroRNAs (miRNAs) are a family of short non-coding RNAs that negatively regulate gene expression at the post-transcriptional level. Hundreds of miRNAs have been found in the human genome and play critical roles in regulating cell signaling pathways such as the transforming growth factor-beta, Wnt, Notch and epidermal growth factor pathways by repressing the expression of different mRNAs expression or through co-regulation with transcription factors [6–9]. Consequently, dysfunction of miRNAs and their target genes can lead to a variety of disorders. Therefore studying the role of miRNAs will provide a better understanding of the molecular events involved in diverse biological processes, and contribute to the identification of new targets in tumor prevention and treatment.

MiRNA-149 directly targets the 3’-UTR of MyD88 mRNA and post-transcriptionally regulates MyD88 protein expression. MiRNA-149 may be a key modulator in the TLR/MyD88 signaling pathway in macrophages through negative regulation of MyD88-dependent Toll-like receptor signaling [10]. In our previous study, the expression of MyD88 was closely associated with paclitaxel resistance in lung cancer A549 cells [11].

The relationship between miRNA-149 expression and paclitaxel chemoresistance in human ovarian cancer cells remains largely unknown. In this study, we investigated whether miRNA-149 modulates cellular sensitivity to paclitaxel by regulating the expression of MyD88 in ovarian cancer A2780 cells.

## Materials and methods

### Cell line and maintenance

The A2780 cell line was obtained from the Institute of Cell Biology (Shanghai, China). The cells were maintained in RPMI 1640 medium (Gibco-BRL, Carlsbad, CA, USA) supplemented with 10 % fetal bovine serum (FBS), 100 U/ml penicillin and 100 U/ml streptomycin at 37 °C in a humidified incubator with 5 % CO_2_. The cells were demonstrated to be free of mycoplasma.

### Construction of miRNA-149 inhibitor and MyD88 lentiviral vectors

MiRNA-149 inhibitor (5’-GGGAGUGAAGACACGGAGCCAGA-3’) was inserted into the LV3-pGLV-H1-GFP/puro lentiviral vector, and siRNA (5’-UUCUCCGAACGUGUCACGUdTdT-3’) was used as a negative control. MyD88 whole cDNA synthesized by GenePharma (GenePharma, Shanghai, China) was subcloned into the LV5-pGLV-EF1a-GFP/Puro Lentiviral plasmid vector. Lentiviruses expressing inhibitor against miRNA-149, MyD88, and the controls were produced by co-transfection of 293 T cells using polybrene (GenePharma, Shanghai, China) according to standard protocols. A2780 (5 × 10^4^) cells were infected with lentivirus at a MOI (multiplicity of infection, pfu number/cell) of approximately 100 for 24 h. Cells were then transferred into complete medium.

### RNA isolation and reverse transcription polymerase chain reaction (RT-PCR)

Small RNAs were purified from differently treated A2780 cells using an RNA purification kit (TIANGEN Biotech, Beijing, China). Total RNA was extracted with Trizol reagent according to the protocol described by the supplier (TakaRa, Dalian, China). First-strand cDNA was synthesized from 1 μg of total RNA in a 20-μl reaction mixture using the PrimeScript RT reagent kit (TakaRa, Dalian, China). Quantitative real-time PCR-based gene expression analysis was performed on a real-time PCR instrument (7300, Step One Plus, Applied Biosystems, USA) using a standard SYBR-Green PCR kit. The parameters used for all PCR reactions were as follows: One cycle of 95 °C for 2 min, followed by 40 cycles of 95 °C for 15 s, and 60 °C for 30 s. Specific primer sets were used for RT-PCR of the U6 control, miR-149, β-actin control, and MyD88. The relative expression of each target gene was calculated using the 2^-ΔΔct^ method.

### Analysis of apoptosis

The percentage of apoptotic cells was quantitated using the Annexin V-PE Kit (Becton-Dickinson) according to the manufacturer's instructions. Stained cells were analyzed by flow cytometry during the first 30 min of staining. ≥10,000 cells were measured using a FACScan instrument (Becton-Dickinson) and the data were analyzed using FlowJo software (Tree Star Inc.).

### Cytotoxicity assay

Cell viability was assessed using the Cell Counting Kit 8 (CCK-8) (Dojindo Laboratories, Kumamoto, Japan) assay. 6 × 10^4^ A2780 cells (100 μl) were seeded into 96-well plates. After 24 h of incubation, paclitaxel (Sigma) was added at concentrations of 0 μM, 0.1 μM, 0.2 μM, 0.3 μM, 0.4 μM and 0.5 μM (each concertration were done repeat five experiments); the cells were then incubated for 48 h. After 48 h, 10 μl of CCK-8 solution was added to each well, followed by 4 h of incubation at 37 °C. The OD values were read at dual wave lengths of 450 nm and 630 nm to determine cell viability using a microplate reader (Thermo Fisher Labsystems).

### Analysis of cell cycle

Cells (2 × 10^6^) were pelleted by spinning for 5 min at 1000 rpm and 4 °C and resuspended in 1 ml of cold PBS. After fixation by adding 4 ml of absolute ethanol, the cells were centrifuged and resuspended in 1 ml of PBS. Then, 100 μl of 200 μg/ml DNase-free RNaseA was added to the cell suspension and incubated for 30 min at 37 °C. The cells were stained with 100 μl of 1 mg/ml propidium iodide (light sensitive) and incubated for 5–10 min at room temperature before analysis.

### Western blot

The protein concentration of each sample was determined by BCA Protein Assay Kit (Beyotime, Jiangsu, China). Equal amounts of protein were loaded and separated discontinuously on 12 % sodium dodecyl sulfate-polyacrylamide gels (SDS-PAGE), and subsequently transferred onto a PVDF membrane (Amersham Pharmacia, UK). The membrane was then incubated in TBST blocking solution (Tris-buffered saline including 0.1 % Tween-20) containing 5 % skim milk for 2 h at room temperature, followed by separate incubation with primary antibodies against Bax, MyD88, Bcl-2 (Cell Signaling, USA) and β-actin (Beyotime, Jiangsu, China) overnight at 4 °C. After washing, the membrane was incubated with HRP-conjugated anti-mouse, anti-rabbit, or anti-goat secondary antibodies for 2 h. After several washes, the immunoblot was detected with enhanced chemi-luminescence reagent (Pierce Biotechnology, USA) according to the manufacturer's instructions.

### Immunocytochemistry

Expression of MyD88 protein was determined using immunocytochemistry. In total, 1 × 10^5^ cells were seeded into a Millicell EZ Slide (Millipore, Shanghai, China). After 24 h of incubation, cells were fixed on slides using 4 % paraformaldehyde. The cells were permeabilized for 10 min with 0.1 % Triton X-100 in PBS and blocked with blocking buffer (2 % BSA, 0.1 % Triton X-100) for 30 min at room temperature. After blocking, the cells were washed with PBS and incubated overnight with rabbit anti-human MyD88 antibodies (Sigma, Shanghai, China) at 4 °C. On the following day, the cells were washed three times with PBS, and then incubated with Cy3-labeled goat anti-rabbit IgG (H + L) (Beyotime, China) for 1 h followed by washing with PBS three times.

### Cell migration assay

Cell migration were evaluated using the Transwell Permeable Support (Corning) according to the manufacturer’s instructions. Five 200-multiple microscopic fields were randomly selected to calculate the total count of the invaded or migrated cells. All assays were conducted three times.

### Statistical analysis

For all analyses, the measurements obtained from the groups were expressed as the means ± SD for all data determined. Statistical analysis was performed using an unpaired Student's t-test followed by Tukey's test. P < 0.05 was considered statistically significant.

## Results

### Decreased sensitivity of A2780 cells to paclitaxel treatment after down-regulation of miRNA-149

**Fig. 1:**
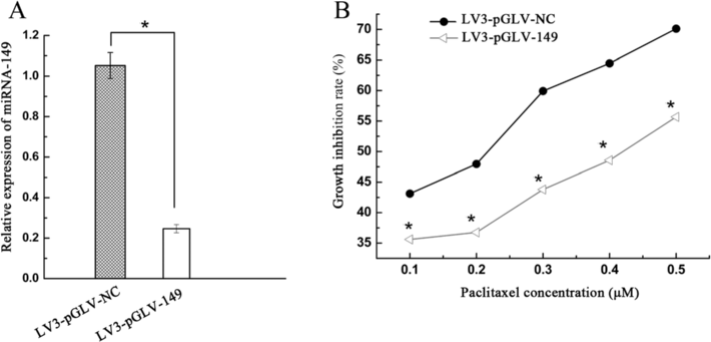
Inhibition of cell growth by paclitaxel was determined in A2780 cells after miRNA-149 knockdown. Paclitaxel treatment was conducted for 48 h with the indicated concentrations. **a** The expression level of miRNA-149 was measured using quantitative real-time PCR in different groups. Asterisk refers to p < 0.01. **b** The inhibition of cell growth by paclitaxel was determined in A2780 cells after miRNA-149 down-regulation. Paclitaxel treatment was performed for 48 h with the indicated concentrations

We used a lentiviral vector expressing inhibitor against miRNA-149 to achieve down-regulation of miRNA-149 in ovarian cancer A2780 cells. Real-time PCR analysis showed that the expression of miRNA-149 in cells transduced with inhibitor to miRNA-149 was markedly lower than that of control A2780 cells (Fig. 1a). In the presence of paclitaxel, the proliferation of A2780 cells was significantly inhibited in the control (Fig. 1b), suggesting that down-regulation of miRNA-149 decreased the sensitivity of A2780 cells to paclitaxel treatment.

### Down-regulation of miRNA-149 increases the expression of MyD88 in A2780 cells

**Fig. 2:**
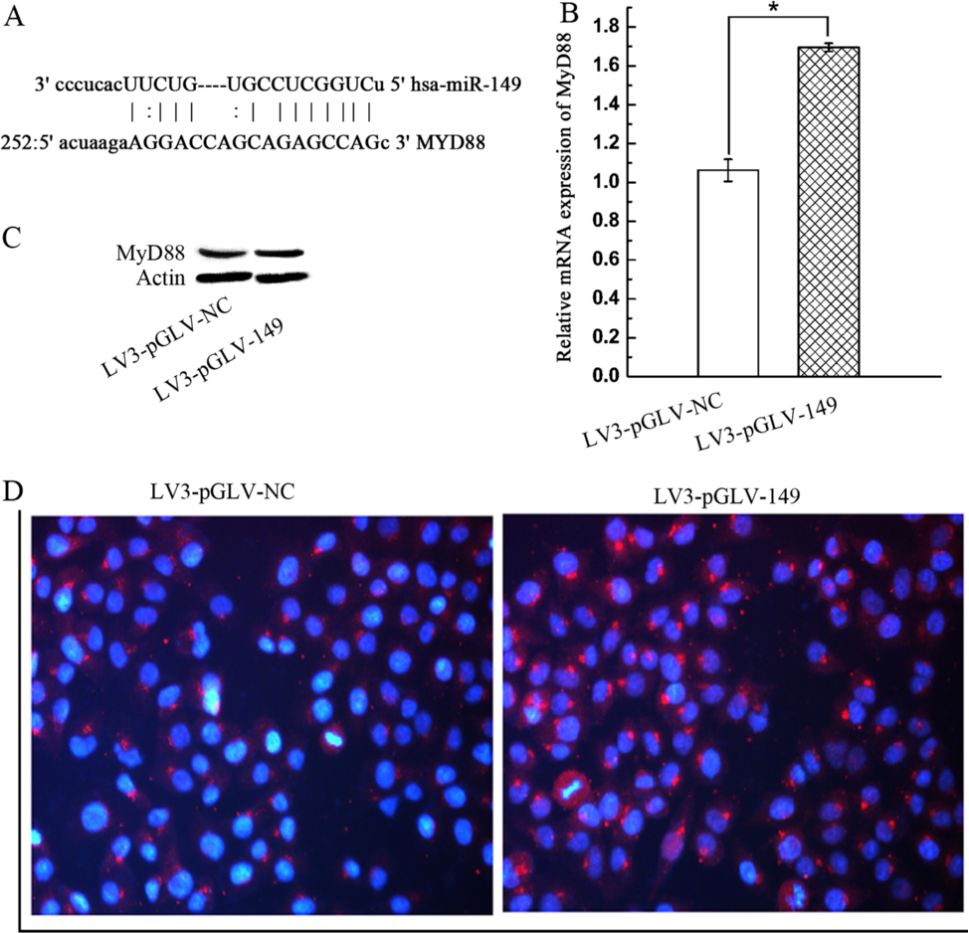
Down-regulation of miRNA-149 increased MyD88 expression in A2780 cells. **a** Seguence of potential binding site of miRNA-149 in the 3’-UTR of MyD88 mRNA. **b** The mRNA level of MyD88 was measured using quantitative real-time PCR in different groups as indicated. Asterisk refers to p < 0.01. **c** MyD88 protein expression was determined by western blot. **d** MyD88 protein expression was determined by immunocytochemistry. Red indicates staining of MyD88

Using miRanda (http://www.microrna.org/) and TargetScan (http://www.targetscan.org/) to analysis the miRNA-149 target sites of MyD88 (Fig. 2a). To assess whether miRNA-149 is involved in the regulation of MyD88 expression in A2780 cells, the expression of MyD88 was evaluated using both real-time PCR, western blot and immunocytochemistry. MiRNA-149 was capable of altering MyD88 expression at the mRNA level in these cells and consequently was able to up-regulate MyD88 mRNA expression by 1.67 fold as compared to the negtive control (Fig. 2b). Furthermore, the overexpression of MyD88 was verified by western blot and immunohistochemistry (Fig. 2c and d). Collectively, the data indicate that miRNA-149 has the potential to regulate MyD88 expression in ovarian cancer cells.

### Decreased sensitivity of A2780 cells to paclitaxel treatment after over-expression of MyD88

**Fig. 3:**
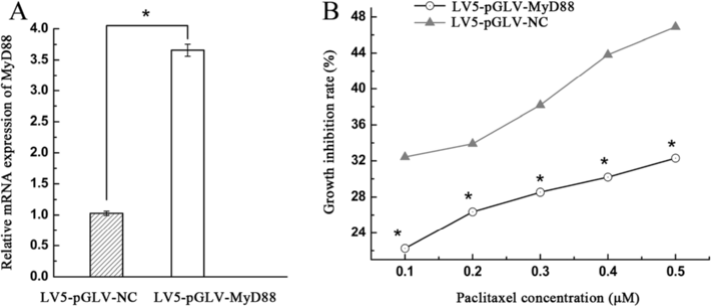
Inhibition of cell growth by paclitaxel was determined in A2780 cells after over-expression of the MyD88 gene. Paclitaxel treatment was conducted for 48 h with the indicated concentrations. **a** The mRNA level of MyD88 was measured using quantitative real-time PCR in different groups (experimental vs. NC control). **b** The inhibition of cell growth by paclitaxel was determined in A2780 cells after MyD88 overexpression. Asterisk refers to p < 0.01

To determine the effects of elevated MyD88 expression in ovarian cancer, MyD88 was over-expressed in ovarian cancer A2780 cells using a lentiviral vector. Over-expression was confirmed by real-time PCR (Fig. 3a). MyD88-overexpressing cells were refractory to growth inhibition by paclitaxel when compared to the control (Fig. 3b).

### Effects of miRNA-149 down-regulation on the cell cycle of A2780 cells

**Fig. 4:**
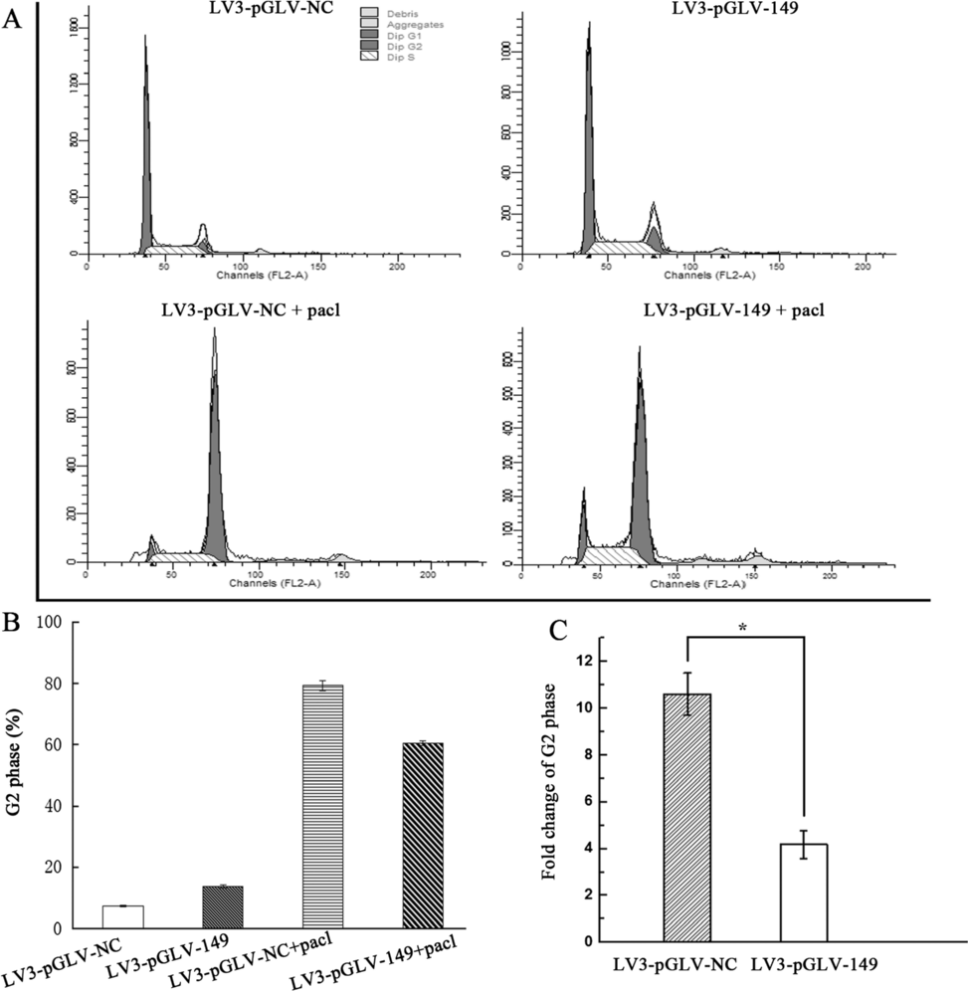
Cell cycle analysis of A2780 cells treated with paclitaxel in relation to miRNA-149 down-regulation. **a** Flow cytometric analysis of the cell cycle with miRNA-149-down-regulated and control cells. **b** G2 phase analysisof the cell cycle with miRNA-149-down-regulated and control cells. **c** Quantitative analysis of the fold change of the G2 phase ratio after paclitaxel treatment in A2780 cells with down-regulation of miRNA-149. Fold change of G2 phase was calculated as: Fold change of G2 phase = G2 phase with paclitaxel treatment/G2 phase without paclitaxel treatment

It is well known that paclitaxel arrests the cell cycle at the G2 phase. To analyze the impact of miRNA-149 expression on paclitaxel’s interference with the cell cycle in A2780 cells, DNA flow cytometric analysis was performed to determine the effect of paclitaxel on the cell cycle of A2780 cells with different expression levels of miRNA-149. A G2 phase increase was found in A2780 cells with miRNA-149 down-regulation and NC cells after treatment with paclitaxel (Fig. 4a and b). The G2 phase fold change was significantly decreased in cells with miRNA-149 down-regulation (Fig. 4c).

### Regulation of apoptosis by miRNA-149 down-regulation in A2780 cells

**Fig. 5:**
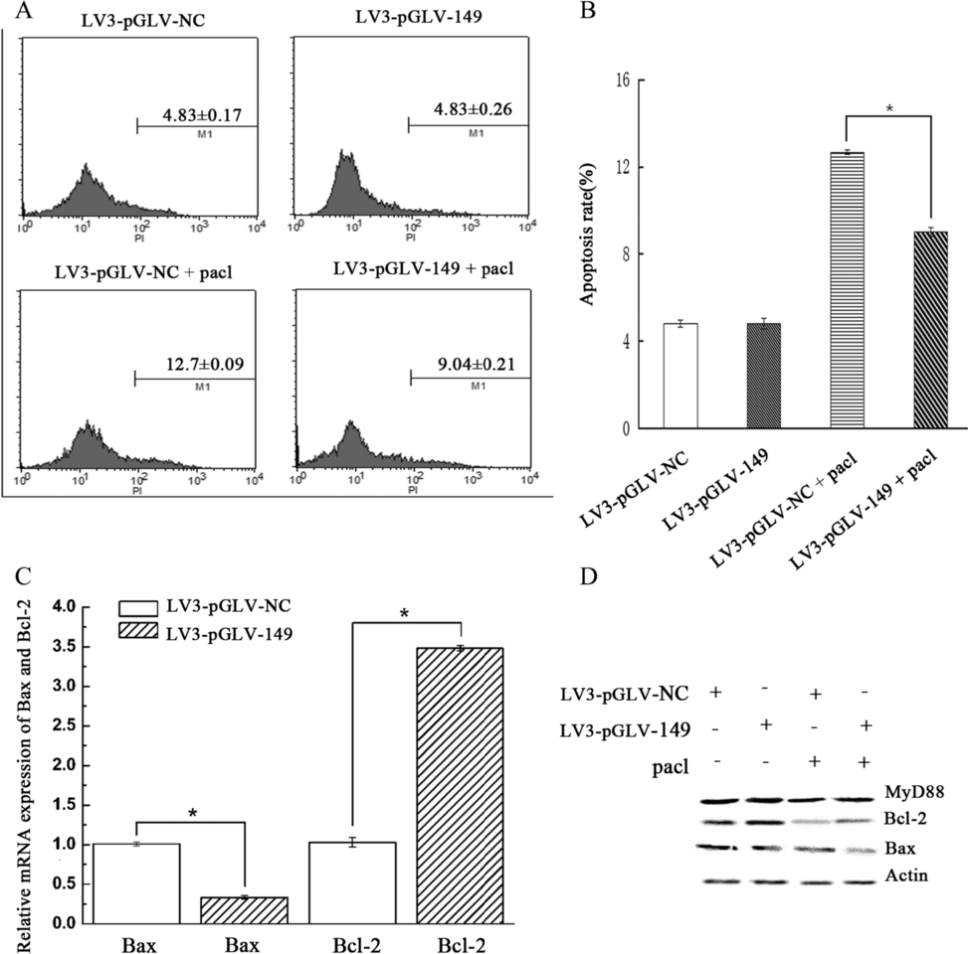
Analysis of apoptosis in A2780 cells with miRNA-149 down-regulation. **a** Apoptosis in miRNA-149-down-regulated A2780 cells was analyzed by Annexin V-PE staining after treatment with paclitaxel. Data are presented as the mean ± SD of three independent experiments. **b** Apoptosis rates of miRNA-149-down-regulated and control cells. Asterisk refers to p < 0.01. **c** Real-time PCR analysis of Bax and Bcl-2 mRNA expression. The results were normalized to the amount of β-actin. Each value represents the average of 3 independent experiments. Asterisk refers to p < 0.01. **d** Western blot analysis of Bcl-2, Bax and MyD88 proteins expression in each group. β-actin expression served as the loading control

Cells were treated with paclitaxel for 48 h prior to the measurement of apoptotic cells with Annexin V. As shown in Fig. 5a and b, a significant decrease in the percentage of apoptotic cells was observed in A2780 cells with miRNA-149 down-regulation (P < 0.01). Down-regulation of miRNA-149 decreased the expression of Bax in A2780 cells and increased the expression of Bcl-2 (Fig. 5c and d). These results suggest that miRNA-149 may be associated with the cell apoptosis induced by paclitaxel in A2780 cells.

### Down-regulation of miRNA-149 promotes the migration of A2780 cells

**Fig. 6:**
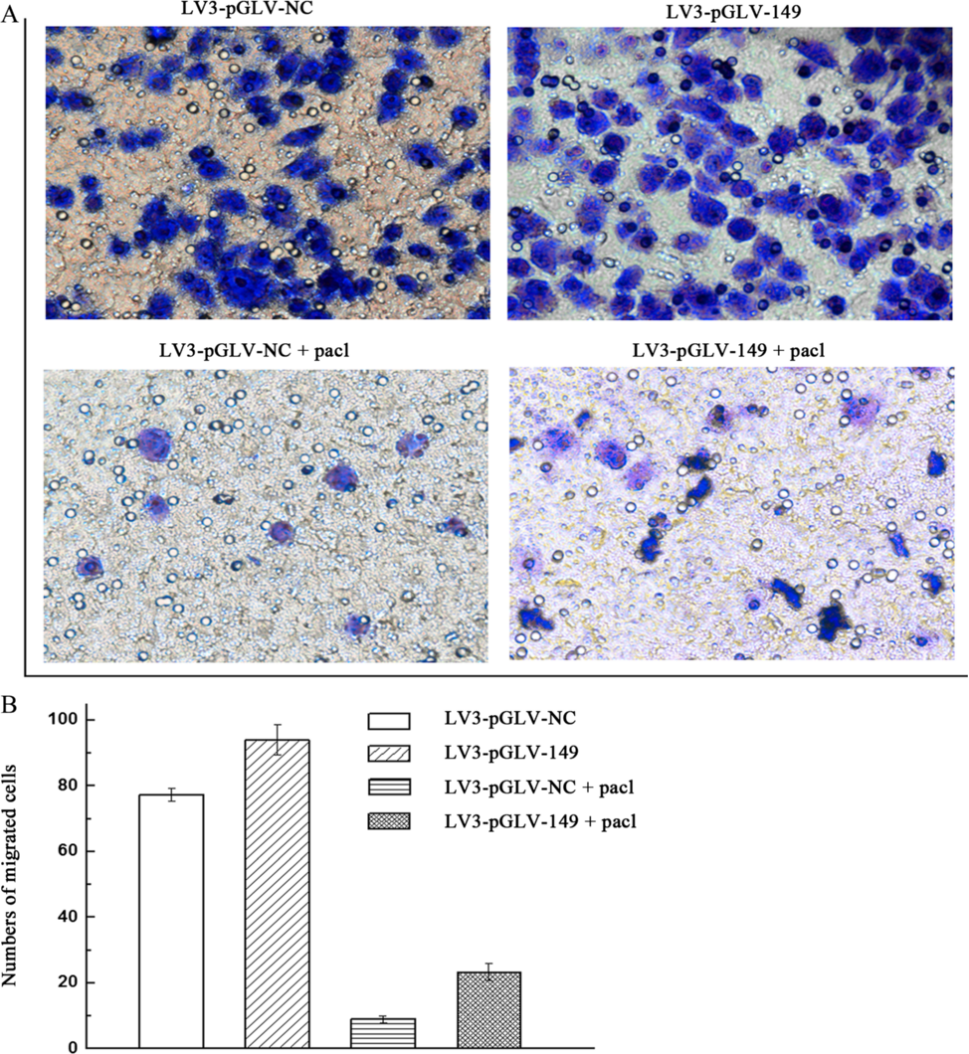
Down-regulation of miRNA-149 promotes A2780 ovarian cancer cell migration in vitro. **a** Cell migration assays were performed on A2780 cells with down-regulation of miRNA-149 and control cells. **b** Migrated cells were visualized by staining with crystal violet and quantitated using a cell counter. Asterisk refers to p < 0.01

As shown in Fig. 6a, A2780 cells migrated faster when miRNA-149 was down-regulated, as indicated by the number of migrated cells within a fixed time (Fig. 6b). These data suggest that down-regulation of miRNA-149 may promote the migration of A2780 cells.

## Discussion

An increasing number of studies suggests that miRNAs play important roles in diverse biological processes, such as development, cell proliferation, apoptosis, fat metabolism, and oncogenesis [12]. Over the years, attention has focused on the role of miRNAs in reversing drug resistance and regulating the sensitivity of cancer cells to chemotherapeutants [13, 14]. Dysregulated expression of various miRNAs has been found in ovarian carcinoma. Dysregulation of the microRNA let-7e and down-regulation of miR-30c, miR-130a, and miR-335 indicate direct involvement of some miRNAs in the development of chemoresistance [15]. Down-regulation of miR-21 promotes apoptosis and chemosensitivity in ovarian cancer [16]. MiRNA-370 [17], miRNA-93 [18], and miRNA-100 [19] are related to the chemosensitivity of ovarian cancer.

Aberrant expression of miR-149 has been reported in many cancers including colorectal cancer [20], nasopharyngeal carcinoma [21], clear-cell renal cell carcinoma [22], prostate cancer [23], and gastric cancer [24]. Li et al. [25] found that over-expression of miR-149 inhibited glioblastoma cell proliferation and migration. As a methylation-sensitive miRNA, miR-149 plays an important role as a tumor suppressor in colorectal cancer with prognostic and therapeutic implications [26]. However, the relationship between miRNA-149 expression and the sensitivity to paclitaxel in ovarian cancer remains unknown. Our results show that down-regulation of miRNA-149 decreases the sensitivity of A2780 cells to paclitaxel treatment.

MyD88 is a key molecular cohesion molecule in the Toll-like receptor (TLR) signaling pathway. Elevated MyD88 expression has been found in parenchymal cells in various types of cancer [27–29]. Expression of MyD88 was elevated in more than 70 % of patients with EOC and has been considered as an indicator of tumor metastasis and paclitaxel chemoresistance, in addition to a factor for significantly poor prognosis [30, 31]. Overexpression of MyD88 decreases the sensitivity to paclitaxel in ovarian cancer and hepatocellular carcinoma cells [32, 33].

In the present study, expression of miRNA-149 in A2780 cells was decreased by specific inhibitor using lentiviral vectors. miRNA-149 down-regulation resulted in increased expression of MyD88 in A2780 cells. The results are consistent with previous results that over-expression of miR-149 in RAW264.7 cells was associated with decreased MyD88 at the protein level [10]. In the presence of paclitaxel, the proliferation of A2780 cells was significantly inhibited in the control group, suggesting that miRNA-149 down-regulation may decrease the sensitivity of A2780 ovarian cancer cells to paclitaxel treatment. Furthermore, the sensitivity to paclitaxel was decreased in MyD88 over-expressing A2780 cells.

Paclitaxel is an effective anti-cancer drug against a variety of cancers. Paclitaxel reduces the dynamicity of the mitotic spindle, causing G2/M cell cycle arrest [34]. In our study, we analyzed the cell cycle of A2780 cells with miRNA-149 down-regulation after treatment with paclitaxel for 48 h. The G2/M phase transition was decreased significantly in A2780 cells in the transduced group compared to the control group. The reduction in the number of cells at G2 phase and the accumulation of cells at the G0/G1 phase suggest that miRNA-149 is associated with paclitaxel chemosensitivity.

Depending on the cell type and drug, cells undergo apoptosis during mitotic arrest or abnormal mitosis [35]. Paclitaxel directly induces apoptosis of several types of tumor cells through a variety of mechanisms, such as phosphorylation of Bcl-2, activation of caspase-3 and caspase-9, and the mitogen-activated protein kinase signal transduction pathway [36–38]. In the present study, flow cytometric assays were used to detect the apoptosis of A2780 cells after down-regulation of miRNA-149. We found that down-regulation of miRNA-149 decreased the apoptosis induced by paclitaxel when compared to the control group. Furthermore, we showed that down-regulation of miRNA-149 in A2780 cells enhanced the expression of the anti-apoptotic protein Bcl-2 and inhibited the expression of the pro-apoptotic protein bax, which may have led to paclitaxel resistance. Our previous study demonstrated that [11] MyD88 was involved in anti-apoptosis and drug resistance through the alteration of bax and bcl-2 levels.

In this study, we have shown that reduced expression of miRNA-149 enhanced the migration of A2780 cells. Chan et al. showed that down-regulation of microRNA-149 suppressed cell migration/invasion and metastasis in breast cancer by targeting migration and invasion-related genes [39]. Taken together, our findings suggest that miR-149 facilitates the migration of ovarian cancer cells through regulation of MyD88.

## Conclusions

Our data demonstrate that miRNA-149 is associated with the cellular response to paclitaxel through regulation of MyD88 in ovarian cancer cells. These findings provide novel insights into the role of miR-149 in the chemosensitivity of human ovarian cancer and indicate that miR-149 could be a putative drug target for therapeutic intervention.
